# Scrotal Abscess in a Patient With Chronic Kidney Disease and a Chronic Foley Catheter: A Case Report

**DOI:** 10.7759/cureus.78677

**Published:** 2025-02-07

**Authors:** Chukwuemeka E Ogbu, Kevin Le, Stella C Ogbu, Toyin Ingram, Byron Ingram

**Affiliations:** 1 Internal Medicine, Cape Fear Valley Health, Fayetteville, USA

**Keywords:** ckd, foley catheter, scrotal abscess, scrotal swelling, uti

## Abstract

Chronic Foley catheter use significantly increases the risk of scrotal abscesses, particularly in patients with comorbidities that impair immune function, such as diabetes and chronic kidney disease (CKD). We report a case of a 56-year-old male with type II diabetes, CKD, and a chronic indwelling Foley catheter who presented with progressive scrotal swelling and pain. Cultures identified *Escherichia coli* and *Serratia marcescens* from both urine and abscess fluid, confirming a possible urinary tract infection (UTI) as the source of scrotal abscess. The patient was found to have an abscess approximately 5 cm in size with significant drainage of abscess fluid overlying necrotic tissue of the right hemiscrotum. The management involved surgical debridement and excision of all necrotic scrotal tissue down to viable tissue, along with abscess washout to reduce the risk of further infections, and culture-directed antibiotic therapy. Outpatient and inpatient physicians, as well as advanced care providers, should prioritize proactive measures that include regular catheter care protocols to reduce the likelihood of complications arising in this vulnerable population.

## Introduction

A scrotal abscess is a surgical emergency that can present and complicate conditions like acute testicular torsion, acute epididymo-orchitis, and acute appendicitis [1,2]. Chronic Foley catheter use is a known risk factor for catheter-associated urinary tract infections (CAUTIs), accounting for a significant proportion of hospital-acquired infections [3]. These infections can extend to the scrotal area, leading to abscess formation, particularly in immunocompromised patients like those with chronic kidney disease (CKD) and type II diabetes mellitus (DM) [4,5]. The morbidity associated with scrotal abscesses is considerable, with complications including overt sepsis and Fournier's gangrene, which can profoundly affect a patient's quality of life [6,7]. Herein, we report the case of a patient with type II DM and advanced CKD, on a chronic Foley catheter, who developed a scrotal abscess. In CKD, immune dysfunction increases the risk of bacterial colonization and infection in the urinary tract, which can spread to the perineal region through lymphatic or direct pathways [5]. Chronic Foley catheterization also worsens this risk by providing a direct conduit for bacterial ascent and bladder colonization [3]. Our case report highlights the importance of early recognition of scrotal abscess in this vulnerable population as delays in diagnosis can lead to life-threatening complications such as Fournier's gangrene. Our case emphasizes the need for a multidisciplinary approach involving urology, infectious disease (ID), and wound care teams to ensure improved outcomes in disease management. In addition to aggressively treating scrotal abscesses, preventing scrotal abscesses in patients with chronic Foley catheters requires routine catheter care, timely catheter changes, and avoiding unnecessary catheterization. Educating patients and caregivers about proper hygiene and recognizing early signs of a urinary tract infection (UTI) should form the base of outpatient primary care visits in these patients.

## Case presentation

The patient was a 56-year-old male with a past medical history of type II DM, benign prostatic hyperplasia (BPH), CKD stage 3a, bilateral hydronephrosis with prior bilateral stent placement, and urinary retention with a chronic indwelling catheter in place for over one month prior. The patient presented to the emergency room with complaints of progressively worsening scrotal pain and swelling in bilateral testicles for the last two days. His medical history was notable for stage 3a CKD, with an estimated glomerular filtration rate (eGFR) of 45 mL/min/1.73 m² and bilateral hydronephrosis secondary to ureteral obstruction, which had been managed with placement of a ureteral stent. He had noticed swelling around his right testicle and dark red urine coming out of the indwelling catheter. The patient had been on the chronic Foley catheter due to persistent urinary retention secondary to his BPH and partial ureteral obstruction, which required prolonged Foley catheter use for bladder decompression. Six months prior to admission, the patient had completed transurethral resection of the prostate (TURP) for BPH, at which time he was noted to have bilateral hydronephrosis as seen on computed tomography (CT) of the abdomen/pelvis with thickening of the bladder wall also noted. He had bilateral ureteral stents and an indwelling catheter in place to manage the hydronephrosis and urinary retention. A renal biopsy was performed, which showed no significant pathology at the time. Per chart review, Foley catheterization was recommended by urology after TURP to prevent complications from overflow incontinence and renal function deterioration from bilateral hydronephrosis.

On initial examination, the patient was afebrile with stable vital signs: blood pressure of 115/80 mmHg, heart rate of 88 bpm, respiratory rate of 18 breaths per minute, temperature of 36.4°C (97.6°F), body mass index of 22.89 kg/m^2^, and oxygen saturation of 100% on room air. Physical examination of the genitalia revealed an edematous scrotum bilaterally, with both testes enlarged. The right testicle had a firm, solid mass palpable on the inferior aspect that was fluctuant in consistency. There was tenderness to palpation of the scrotal contents and erythema that extended to the perineum bilaterally. No crepitus was noted, and digital rectal examination was unremarkable with no prostate tenderness or enlargement.

Laboratory studies were significant for leukocytosis, elevated C-reactive protein (CRP), and erythrocyte sedimentation rate (ESR). Serum creatinine was elevated from a baseline of 2.0 mg/dL, with reduced eGFR. As noted in Table 1, blood glucose was elevated with a hemoglobin A1C level of 12.6 (reference range: <7.0%). The basic metabolic panel was significant for mild hyponatremia, mild hyperkalemia, hyperphosphatemia, elevated anion gap, and hyperglycemia, as shown in Table 1.

**Table 1:** Admission lab values

Lab	Patient’s value	Reference range
White blood cell count (WBC)	15,200/mm^3^	4,500-12,500/mm^3^
C-reactive protein (CRP)	6.5 mg/L	<5 mg/L
Erythrocyte sedimentation rate (ESR)	50 mm/hr	<15 mm/hr
Serum creatinine	9.0 mg/dL	0.60-1.30 mg/dL
Estimated glomerulus filtration rate (eGFR)	6.3 mL/min/1.73 m^2^	>60 mL/min/1.73 m^2^
Sodium	128 mmol/L	136-145 mmol/L
Potassium	5.7 mmol/L	3.4-4.9 mmol/L
Chloride	97 mmol/L	98-107 mmol/L
Phosphorus	7.1 mmol/L	2.5-4.5 mmol/L
Anion gap	18 mmol/L	1-11 mmol/L
Glucose	299 mg/dL	74-106 mg/dL
Hemoglobin A1C	12.6%	<7%

Urinalysis showed 3+ white blood cell esterase, a white blood cell count (WBC) >180, and 2+ bacteria, with orange-colored urine that was densely turbid. Urine chemistries showed negative nitrite and bilirubin. The urine sample from the indwelling catheter was sent for culture, and results showed *Escherichia coli *and *Serratia marcescens*. The methicillin-resistant *Staphylococcus aureus *(MRSA) polymerase chain reaction (PCR) of the nares was positive. *Treponema pallidum *antibody, *Chlamydia trachomatis*, and *Neisseria gonorrhoeae *PCR were negative.

**Figure 1:**
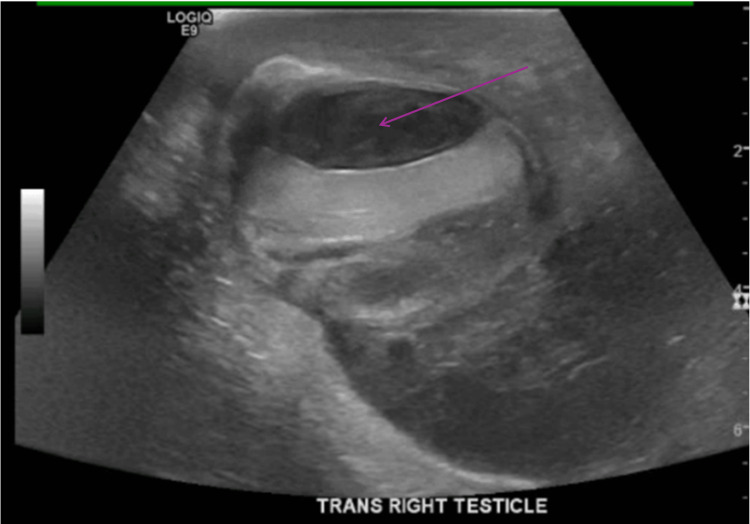
Ultrasound (transverse view) of the right scrotum The arrow shows complex mass/fluid collection.

**Figure 2:**
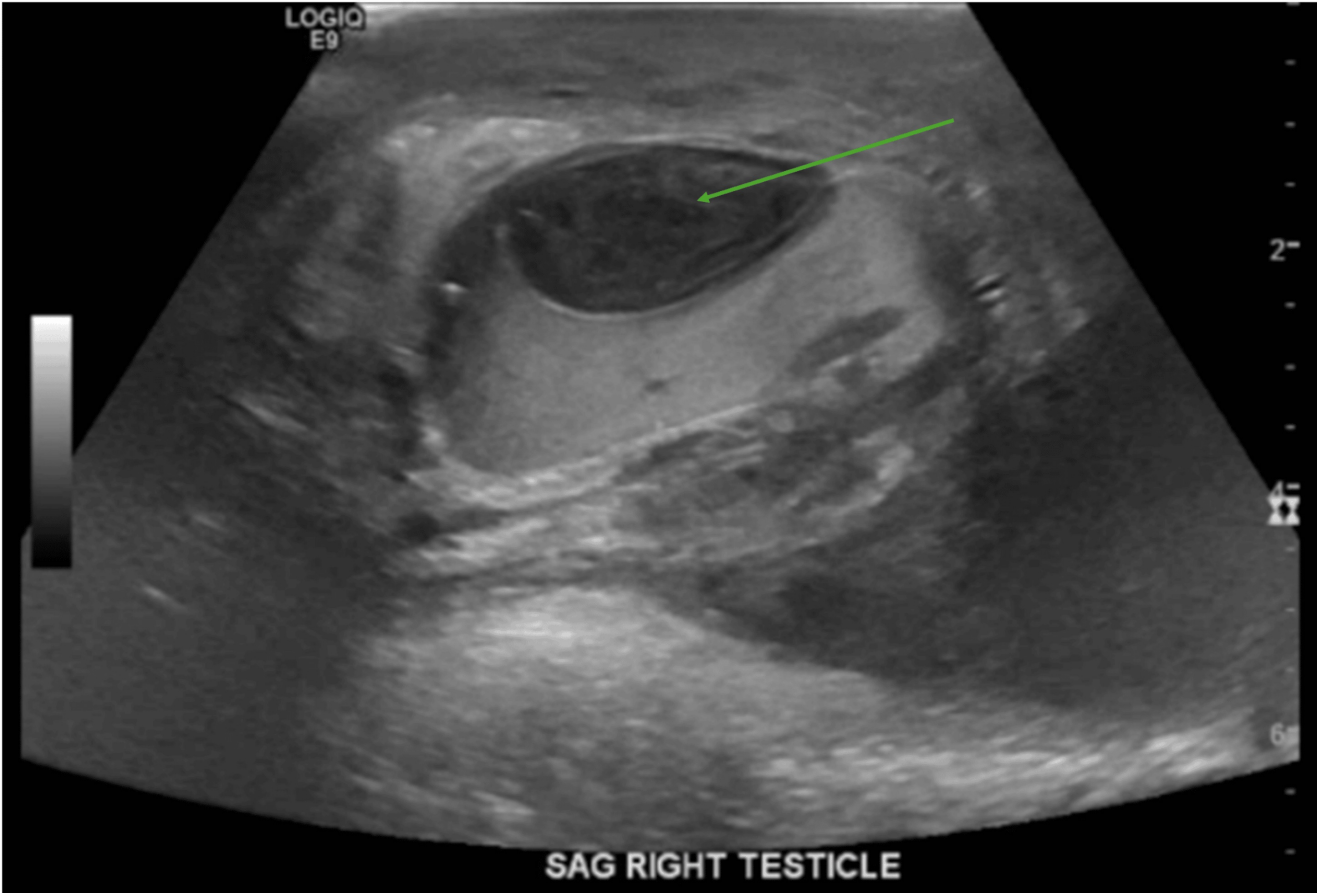
Ultrasound (sagittal view) of the right testis The arrow indicates fluid collection.

Given the clinical presentation, an urgent scrotal ultrasound was performed to evaluate the potential causes of the scrotal mass (Figures 1-2).

The ultrasound showed a 2.9 x1.2x2.8 cm avascular solid mass in the right scrotal subcapsular sac with compression of the right testicular parenchyma and complex fluid surrounding the right testicle. The right testicle measured 4.7x2.2x3.6 cm, and the left measured 4.1x2.0x3.3 cm with hypoechoic complex fluid. There was also evidence of complex fluid surrounding the right testicle, consistent with a large abscess. The left testicle appeared enlarged but without any discrete masses or abscess formation.

**Figure 3:**
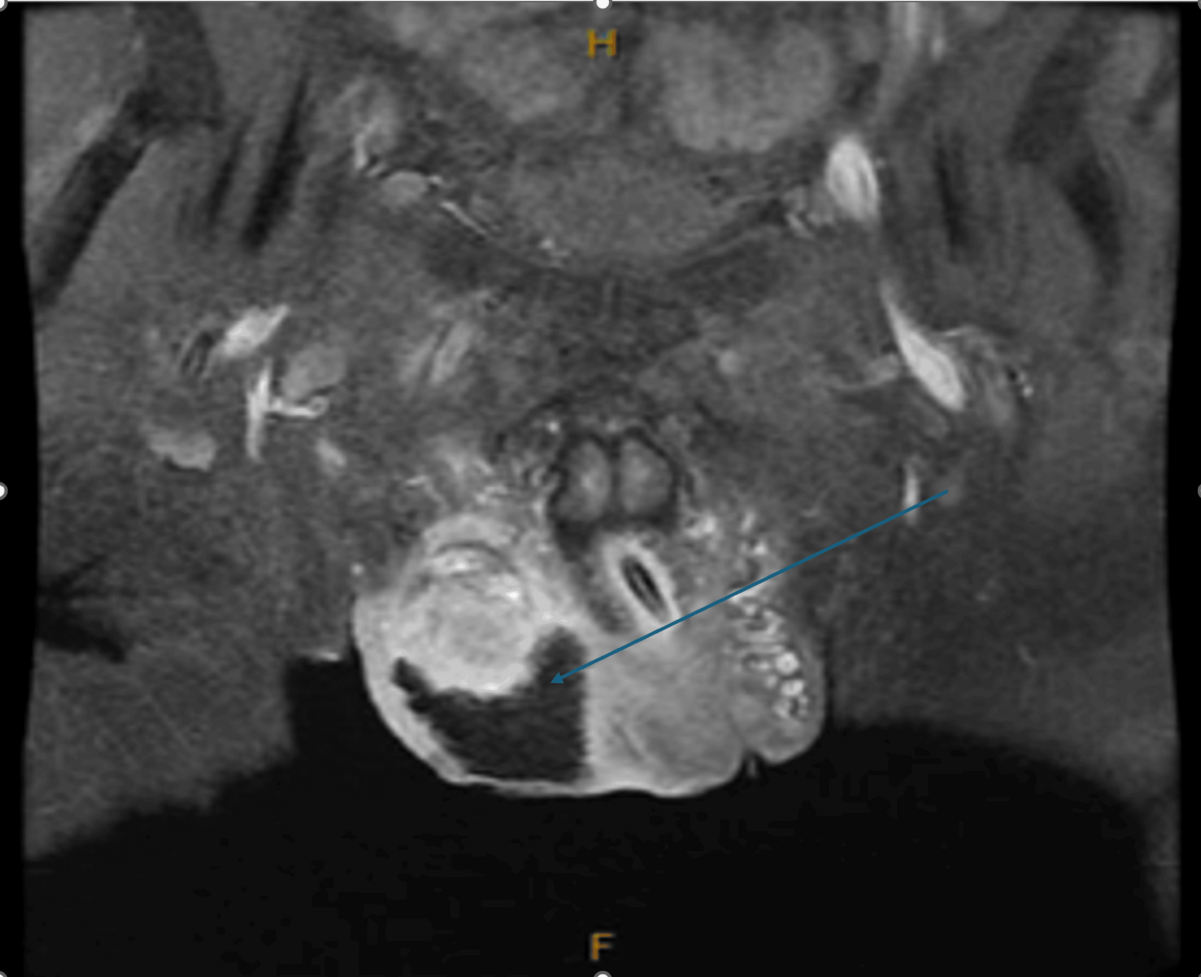
Magnetic resonance imaging on day 1 post-scrotal drainage The arrow indicates some remaining micro-collections/abscesses.

Based on the ultrasound findings and clinical suspicion of a scrotal abscess in the setting of an underlying complicated UTI, the polymicrobial nature of the infection, and the patient's immunocompromised state due to CKD stage 3a, the patient was started on broad-spectrum intravenous antibiotics, including cefepime, metronidazole, and linezolid, to cover both gram-negative and anaerobic organisms and resistant gram-positive bacteria after blood was collected for blood culture. The patient was admitted and promptly seen by the urology team, who recommended scrotal exploration. During the procedure, the patient had a large abscess, approximately 5 cm in size, with significant drainage of abscess fluid overlying necrotic tissue of the right hemiscrotum. Excisional debridement of all necrotic scrotal tissue down to viable tissue was performed and a significant amount of abscess fluid was washed out. The tissue pocket was packed with iodoform gauze and fluffed Kerlix™ (Cardinal Health, Dublin, USA). In addition, the bilateral ureteral stents were removed, and a new indwelling catheter (Teleflex, Morrisville, USA) was placed. Pathology of necrotic scrotum tissue was performed, and there were no signs of malignancy observed. Cultures obtained from the abscess grew *S. marcerens* and *E. coli*. Antibiotic susceptibility testing showed that the organisms were sensitive to the current regimen of cefepime, metronidazole, and linezolid. Magnetic resonance imaging (MRI) of the pelvis, performed after the procedure, showed surgical packing material with lentiform fluid at the anterior right testicle, causing a mass effect on the testicle, measuring 1.3x2.3 cm and suggesting micro-abscess (Figure 3).

Concurrent with the urology consultation, the patient was seen by the ID team, who recommended continuing cefepime, metronidazole, and linezolid therapy for five days. He was subsequently switched to piperacillin/tazobactam and doxycycline for targeted coverage for two weeks. Given the patient's detected MRSA-PCR, empiric antibiotic therapy with linezolid was chosen to cover MRSA. It was also advantageous in the setting of his CKD, as it avoids nephrotoxic side effects compared to vancomycin.

On the third day post-surgery, the Penrose drain (Cardinal Health, Dublin, USA) that was placed on the day of scrotal exploration was removed, and the scrotal wound was left to heal by secondary intention with regular dressing changes. Follow-up laboratory tests showed a decreasing trend in WBC and renal function remained stable without further elevation in serum creatinine, as shown in Table 2.

**Table 2:** Discharge lab values

Lab	Patient’s value	Reference range
White blood cell count (WBC)	6,500/mm^3^	4,500-12,500/mm^3^
C-reactive protein (CRP)	3 mg/L	<5 mg/L
Erythrocyte sedimentation rate (ESR)	12 mm/hr	<15 mm/hr
Serum creatinine	3.6 mg/dL	0.60-1.30 mg/dL
Estimated glomerulus filtration rate (eGFR)	18.8 mL/min/1.73 m^2^	>60 mL/min/1.73 m^2^
Sodium	137 mmol/L	136-145 mmol/L
Potassium	4.4 mmol/L	3.4-4.9 mmol/L
Chloride	101 mmol/L	98-107 mmol/L
Phosphorus	4.5 mmol/L	2.5-4.5 mmol/L
Anion gap	10 mmol/L	1-11 mmol/L
Glucose	138 mg/dL	74-106 mg/dL

The patient remained hospitalized for 28 days due to worsening CKD, which required inpatient dialysis, and delays in securing a post-hospitalization placement at a skilled nursing facility. He completed a further 14-day course of antibiotics while inpatient, with the healing of his scrotal wound by secondary intention. He was advised to continue Foley catheter care and was scheduled for outpatient urology follow-up. Two sets of blood cultures showed no growth, and he remained afebrile during his admission. He was discharged from the hospital with a three-week follow-up with his primary care provider, urologist, and nephrologist.

## Discussion

The literature suggests that Foley catheters significantly increase the risk of developing scrotal abscesses, mainly due to the direct pathway they provide for bacteria to ascend and colonize the urinary tract and surrounding tissues [3]. The case presentation describes a patient with a chronic Foley catheter who developed a scrotal abscess co-infected with *E. coli *and *S. marcescens*. This patient had a history of uncontrolled type II DM, CKD, and bilateral nephrostomy tubes due to ureteral obstruction, and now presented with worsening scrotal swelling. While scrotal swelling is common in such clinical scenarios, it necessitates consideration of differentials like chronic urinary retention, acute epididymitis, testicular torsion, simple cysts, inguinal hernias, or uncomplicated UTIs. However, the presence of systemic infection indicators like elevated WBC and CRP levels points decisively toward a more serious underlying UTI [6]. Therefore, there is a need for physicians to develop a high index of suspicion in patients with chronic Foley catheters, alongside the necessity for immediate and thorough clinical examination and diagnostic workup.

Our patient's urinalysis and subsequent urine and abscess fluid cultures were positive for *E. coli* and *S. marcescens*, suggesting the possibility of an ascending infection facilitated by the chronic indwelling Foley catheter. Imaging via ultrasound and scrotal exploration with washout was necessary to confirm the diagnosis and manage the abscess. Antibiotic susceptibility testing was conducted to determine the appropriate antimicrobial therapy for both organisms, which were found to be susceptible to cefepime, metronidazole, and linezolid. Given the patient's advanced CKD, the choice of antibiotics was guided by the need to avoid nephrotoxic drugs.

This patient's definitive management involved surgical intervention and targeted antibiotic therapy. The surgical approach was crucial in preventing potentially life-threatening complications like Fournier's gangrene or abscess recurrence. It allowed for the complete drainage of the abscess and the removal of any necrotic tissue, reducing the risk of further infection. Furthermore, the isolated organisms, typically associated with UTIs, suggested an ascending infection facilitated by the chronic Foley catheter [3,4,6,7]. Antibiotic treatment was guided by both empirical evidence and culture sensitivities to address the specific pathogens involved, while also considering the patient's underlying renal impairment, which can limit the use of certain nephrotoxic drugs.

One limitation of our case report is its single-patient narrative, which limits generalizability to broader populations. The lack of long-term follow-up data also does not allow for evaluation of potential recurrence, chronic complications, or the impact of preventive measures. Future studies involving larger cohorts of patients with both CKD and chronic Foley catheters or similar comorbidities are necessary to assess long-term outcomes, such as the development of scrotal abscesses, chronic infectious disease burden, and functional recovery. Preventive strategies are also important to minimize the risk of scrotal abscess formation. These include strict adherence to catheter care protocols, regular catheter changes every 2-4 weeks, and proper perineal hygiene [8]. Monitoring for early signs of infection, such as fever or catheter blockage, coupled with prompt intervention using urine cultures and empiric antibiotics, can also reduce progression to abscess formation [8]. Early urologic intervention in patients with recurrent UTIs or evidence of bladder dysfunction can further reduce risks. These preventive measures should be incorporated into routine care for patients with chronic Foley catheters [9].

## Conclusions

Chronic Foley catheter use is a risk factor for scrotal abscesses as it facilitates the ascent of opportunistic pathogens. Underlying disease comorbidities, such as CKD, nephrostomy stents, a prior history of surgery, and diabetes, decrease immunity and enhance the susceptibility to and the severity of ascending infections. Here, we detailed the case of a male patient with a scrotal abscess whose management involved a multidisciplinary approach, leading to the successful resolution of both the abscess and the infection. Timely surgical intervention, which includes incision and drainage, excision, and exploratory debridement, combined with culture-directed antibiotic therapy, is the cornerstone of managing scrotal abscesses. Tailoring antibiotics to local resistance patterns and individual patient comorbidities is important in optimizing outcomes.
